# Superfluid flow above the critical velocity

**DOI:** 10.1038/s41598-017-08941-8

**Published:** 2017-08-22

**Authors:** A. Paris-Mandoki, J. Shearring, F. Mancarella, T. M. Fromhold, A. Trombettoni, P. Krüger

**Affiliations:** 1 Midlands Ultracold Atom Research Centre, School of Physics & Astronomy, University of Nottingham, Nottingham, NG7 2RD United Kingdom; 2 5. Physikalisches Institut and Center for Integrated Quantum Science and Technology, Universität Stuttgart, 70569 Stuttgart, Germany; 3 Nordic Institute for Theoretical Physics (NORDITA), SE-106 91 Stockholm, Sweden; 4 Stockholm University, SE-106 91 Stockholm, Sweden; 5 CNR-IOM DEMOCRITOS Simulation Center, Via Bonomea 265, I-34136 Trieste, Italy; 6 SISSA and INFN, Sezione di Trieste, Via Bonomea 265, I-34136 Trieste, Italy; 7 Department of Physics and Astronomy, University of Sussex, Brighton, BN1 9QH United Kingdom; 8 Present Address: Instituto de Física, Universidad Nacional Autónoma de México, P.O.B. 20-364, Mexico City, 01000 Mexico

## Abstract

Superfluidity and superconductivity have been widely studied since the last century in many different contexts ranging from nuclear matter to atomic quantum gases. The rigidity of these systems with respect to external perturbations results in frictionless motion for superfluids and resistance-free electric current flow in superconductors. This peculiar behaviour is lost when external perturbations overcome a critical threshold, i.e. above a critical magnetic field or a critical current for superconductors. In superfluids, such as liquid helium or ultracold gases, the corresponding quantities are a critical rotation rate and a critical velocity respectively. Enhancing the critical values is of great fundamental and practical value. Here we demonstrate that superfluidity can be completely restored for specific, arbitrarily large flow velocities above the critical velocity through quantum interference-induced resonances providing a nonlinear counterpart of the Ramsauer-Townsend effect occurring in ordinary quantum mechanics. We illustrate the robustness of this phenomenon through a thorough analysis in one dimension and prove its generality by showing the persistence of the effect in non-trivial 2d systems. This has far reaching consequences for the fundamental understanding of superfluidity and superconductivity and opens up new application possibilities in quantum metrology, e.g. in rotation sensing.

## Introduction

The breakdown of superfluidity^1, 2^ or superconductivity^3^ above a critical velocity or a critical current, respectively, is caused by the production and growth of excitations. When the flow velocity of a superfluid exceeds a critical velocity *v*_*c*_ the creation of excitations becomes energetically favourable. This destroys the frictionless motion, as shown in classic experiments with superfluid helium^4^ and in more recent experiments with ultracold bosons^5^ and fermions^6^.

The occurrence of a critical velocity is conventionally understood in terms of a maximum velocity below which there is no or at most a bounded production of excitations. While almost no excitations are present for subcritical velocities, a fast onset of growing excitations occurs for supercritical velocities. The production rate of excitations gradually decreases for further increased velocity as the kinetic energy of the fluid becomes so high that it dominates all other energy scales including those related to defects in the flow channel or in the trapping potential.

**Figure 1:**
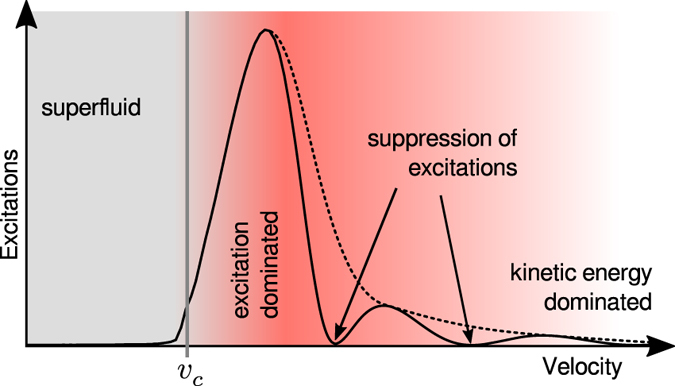
Schematic plot of the qualitative behaviour of excitations in a superfluid. Excitations are plotted as a function of the superfluid velocity at a given time (dotted line: standard picture; solid line: this work). For velocities below a critical velocity (*v* < *v*_*c*_), the production of excitations is suppressed and superflow persists: at longer times the number of excitations remains very small. At higher velocities (*v* > *v*_*c*_) a sharp onset of excitations (growing with time) destroys the superfluid properties. At very high velocities ($v\gg {v}_{c}$) the kinetic energy becomes so large that the defect hardly affects the flow. This standard picture has to be adjusted when resonant quantum interference reinstates superfluidity with fully suppressed excitation growth at a series of discrete supercritical velocities. The solid line shows an example for a rectangular defect shape (for the same plot at different times see Fig. 3). A detailed discussion of how it is possible to quantify the production of excitations in a specific case is provided in the text, see Eq. 3.

This conventional scenario is qualitatively illustrated in Fig. 1 and contrasted to the main result of this paper, namely the presence of supercritical, arbitrarily large, velocities for which there is a bounded growth and production of excitations occurring for a specific class of defects. For these velocities superfluidity occurs due to a resonance between the characteristic length scales of the defect and of the incident wave. This phenomenon can be viewed as a nonlinear counterpart of the Ramsauer-Townsend effect that occurs in the linear case described by the Schrödinger equation^7^. Here we demonstrate this in a case study of the flow of one-dimensional (1d) Bose gases in the presence of a rectangular defect. To illustrate the generality of the effect in terms of dimensionality we then consider 2d defect potentials, showing that the wavepacket dynamics displays the same qualitative results persisting even in the presence of a non-separable potential.

In general, the rate of excitation growth depends on the microscopic details of the superfluid and on how it is coupled to the environment. The details of this coupling determine the dissipation mechanism causing the creation of excitations and, ultimately, *v*_*c*_. In the case of a superfluid moving through a confining channel, for example, the microscopic interactions with the walls provide the source of possible breakdown of superfluidity. In the present paper we treat the case of a superfluid moving with velocity *v* in presence of a single defect at a fixed position that is gradually ramped on.

A simple and ingenious way to estimate *v*_*c*_ and give a qualitative explanation of the breakdown of superfluidity is provided by the Landau criterion^8^. The Landau criterion is a cornerstone of our understanding of the dynamical behaviour of superfluids, stating that superfluid flow is sustained against external perturbations or defects up to a critical value of the velocity^8^. Its elegance, power and usefulness rely both on simplicity and generality. There is no need to know the specific nature of the perturbation or the characteristics of the defects, no need to know all the microscopic details of the superfluid, and no need to compute the excitation spectrum of the moving system; only the knowledge of the low-energy excitation spectrum *ε*(*p*) of the system at rest is required. In Landau’s treatment the microscopic description of the dissipation sources is not considered, since the former relies on the determination of the conditions under which the creation of elementary excitations becomes energetically favourable, in contrast to microscopic computations in which the interactions of the superfluid with its environment are explicitly taken into account. By applying a Galilean transformation to the co-moving frame it can be shown that for *v* < *v*_*L*_ (where ${v}_{L}=\,\min \,\frac{\varepsilon (p)}{p}$ is the Landau critical velocity^8^) the production of elementary excitations is energetically unfavourable. From the Landau criterion it follows that for Bose gases with a weak, short-range interaction, the Landau critical velocity is equal to the sound velocity *c*, so that here “supercritical” means “supersonic”. The detailed analysis of different superfluid systems, including helium and ultracold gases, shows that the Landau criterion often quantitatively overestimates the value of *v*_*c*_, especially in the 2d and 3d cases (see also recent findings in 1d^9^). This, together with the occurrence of a non-vanishing superfluid fraction also above the Landau critical velocity^10^ implies that the Landau criterion is neither necessary nor sufficient. However, despite the fact that in general *v*_*L*_ ≠ *v*_*c*_, the identification of a critical velocity above which the production of excitations destroys the superfluid motion is a criterion of paramount clarity and relevance.

For *v* > *v*_*c*_ superfluidity is destroyed in the sense that a non-vanishing normal fluid component develops and even at zero temperature the superfluid fraction *ρ*_*S*_ is smaller than the total density *ρ*, e.g. resulting, for the case of superconductivity, in a non-vanishing resistance. Superfluidity is *completely* destroyed once *ρ*_*S*_ = 0 is reached. The conventional picture implies that the latter happens for any velocity larger than some finite velocity, which we denote as ${v}_{c}^{\ast }$ to keep it in general distinct from *v*_*c*_ (of course, ${v}_{c}^{\ast }\ge {v}_{c}$).

The Landau criterion is based on a perturbative treatment of “small” defects affecting the superfluid motion. The possibility to explore superfluid motion in a non-perturbative regime of parameters has attracted considerable interest, e.g. in non-perturbative and/or exact studies of the dynamical propagation of a superfluid in presence of “non-small” defects (of tunable shape and intensity) or periodic potentials^11, 12^. The existence of flow in channels above the Landau critical velocity was first discussed in ref. 13, and the density pattern in the supercritical flow of ^4^ *He* has been in discussed in ref. 14.

The point we address in this paper at a non-perturbative level is the surprising possibility of stable superfluid propagation with bounded emission of excitations (i.e. *ρ*_*s*_ = *ρ* at zero temperature) for a series of specific, but arbitrarily large incident velocities. Therefore, the notion of the existence of a finite critical velocity above which no superfluid flow exists can no longer be maintained universally.

## Model

To illustrate the existence of perfect supercritical flow and the associated phenomena arising from it, we choose defects of rectangular shape in the 1d flow of a superfluid, whose behaviour is governed by the Gross-Pitaevskii equation^15^:

1 $$i\hslash \frac{\partial \psi }{\partial t}=-\frac{{\hslash }^{2}}{2m}\frac{{\partial }^{2}\psi }{\partial {x}^{2}}+V(x,t)\psi +g{|\psi |}^{2}\psi $$

where *ψ*(*x*, *t*) is the condensate wavefunction, *g* is the one-dimensional nonlinear coefficient^16^, and *V*(*x*, *t*) is an applied external potential.

**Figure 2:**
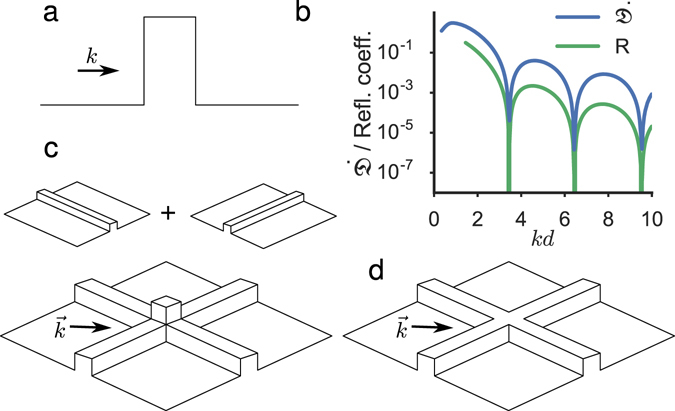
Square-shaped defect potentials. (**a**) Schematic plot of the 1d potential (Eq. 2). (**b**) Comparison of the asymptotic behaviour of $\dot{{\mathfrak{D}}}$ and of the reflection coefficient *R* for *g* = 0 as a function of *kd*. Panels (c) and (d) are depicting the 2d potential (Eq. 6) for *ε* = 2 (separable potential) and *ε* = 1 (non-separable potential), respectively.

We consider a homogeneous system with stationary flow at velocity *v* in an initially flat potential, in which a rectangular defect is then introduced to study the dynamical response. The potential is therefore chosen to be (see Fig. 2a):

2 $$V(x,t)=\{\begin{array}{cc}{V}_{0}\,\tanh ({t}^{{\scriptscriptstyle 2}}/{\alpha }^{{\scriptscriptstyle 2}}) & {\rm{f}}{\rm{o}}{\rm{r}}\,0\, < \,x < d\\ 0 & {\rm{o}}{\rm{t}}{\rm{h}}{\rm{e}}{\rm{r}}{\rm{w}}{\rm{i}}{\rm{s}}{\rm{e}}\end{array},$$

where *d* is the width and *V*_0_ the strength of the defect, which is ramped on at time *t* = 0 with a speed parametrized by *α*. At time *t* = *t*_barrier_ ≡ 1.5*α* the value of the defect is 0.98*V*_0_, so it is almost completely turned on as shown in Fig. 4b. The initial state (when the barrier is absent) *ψ*(*x*, *t* = 0) = *ψ*_0_ *e* ^*ikx*^ is a plane wave with momentum *k* and velocity *v* = *ħk*/*m* corresponding to atom density *n* ≡ |*ψ*_0_|^2^. Since for *t* = 0 the defect is absent, *ψ*(*x*, *t* = 0) is a solution with momentum *k* of the time-independent Gross-Pitaevskii equation. The purpose of ramping on the defect is to adiabatically lead the system towards possible supercritical superfluid solutions for specific values of the momentum *k*.

Our treatment is based on the use of Gross-Pitaevskii equation and we do not include dissipation explicitly. In current experiments with ultracold atoms superfluidity is preserved for long times of up to a few seconds with velocities of ~μm/ms and rings with radius ≳20 μm^17, 18^, while our simulations consider a time span of less than 100 ms for typical experimental parameters (see Experimental Considerations). We therefore expect that the time scale at which superfluidity above the critical velocity is destroyed by dissipation is long compared to the time scales we numerically investigated.

## Methods

From a theoretical perspective, a challenging point is to define the transmission and reflection coefficients in presence of a nonlinearity (*g* ≠ 0). The reason is that the usual definitions used for linear matter waves^19, 20^ do not apply to this case. In fact the superposition principle of an incoming and a reflected wave is no longer valid, and furthermore, bound states in the defect can be present due to the interaction term^21^. For a weakly interacting Bose gas one can quantify the transmitted part of an incident wavepacket by the study of the dynamics in presence of defects^22–29^. A different approach, alternative to defining transmission and reflection coefficients, is to characterise the breakdown of superfluidity by studying the drag exerted by a matter wave on an obstacle^30^ or the stationary wave patterns of a *δ*-like potential moving at supersonic velocity^31^. A special case is represented by rectangular defects in 1d. Even though the difficulty of defining the transmission coefficient for a finite *g* ≠ 0 persists, one can write solutions of the time-independent Gross-Pitaevskii equation in terms of Jacobian functions^32, 33^. Using such wavefunctions the current-phase relation has been determined for subsonic motion^34, 35^. Neglecting the mean-field interaction outside the potential well gives raise to a major formal simplification, since it makes possible to analytically calculate the transport properties of the system in terms of incoming and outgoing waves and resonances and bound states are obtained in closed form^36^.

**Figure 3:**
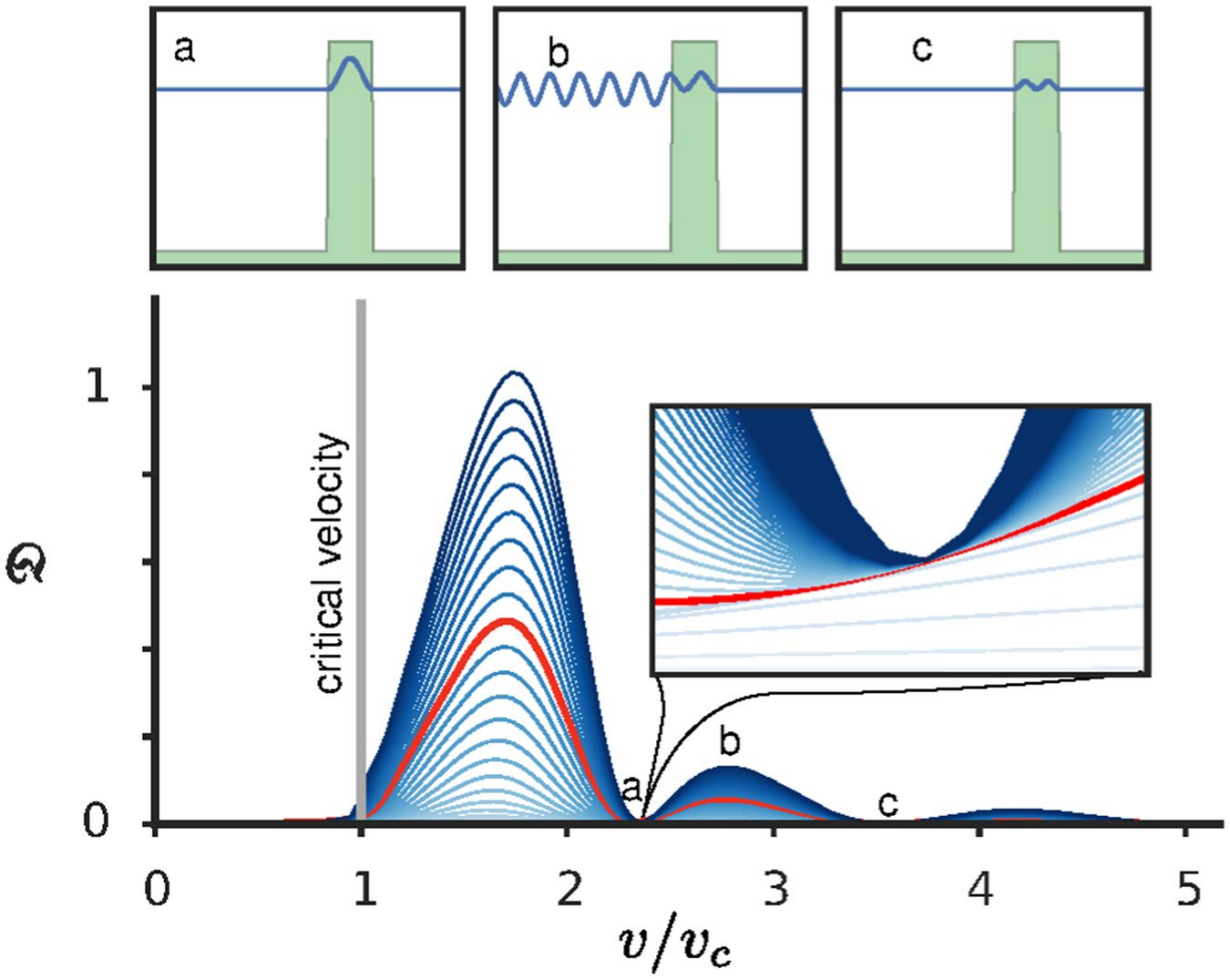
Growth and suppression of excitations. Disturbance ${\mathfrak{D}}$ given by Eq. 3 vs *v*/*v*_*c*_ at various equidistant times (growing from the bottom of the figure), where *v*_*c*_ is equal to the sound velocity *c*. Darker lines indicate longer times. The line highlighted in red corresponds to *t* = *t*_barrier_. At specific values of *v*, the disturbance does not grow with time after the barrier has finished rising. The insets illustrate this phenomenon in detail for the first minimum. Panels (a–c) show the density *n*(*x*) = |*ψ*(*x*, *t*_0_)|^2^ (in blue) and the potential *V*(*x*, *t*_0_) (in green) at time *t*_0_ = 2.2*t*_barrier_ for three different initial values of *v* corresponding to the minimum and maximum points for ${\mathfrak{D}}$ indicated as “a”, “b”, “c” in the main figure. The initial condition is a plane wave travelling with momentum *ħk* = *mv* towards the right. The simulation parameters are *g* = 15 × (*ħ* ^2^/2*md*), *α* = 3 × (2*md* ^2^/*ħ*) and *V*_0_ = 2 × (*ħ* ^2^/2*md* ^2^).

Here we devise another approach where we quantify the production and growth of excitations by introducing the time-dependent quantity ${\mathfrak{D}}(t)$, which we refer to as the *disturbance*, defined as

3 $${\mathfrak{D}}(t)={\int }_{-L}^{0}dx{({|\psi (x,t)|}^{2}-{|\psi (x,t=0)|}^{2})}^{2},$$

where the integral is calculated over the region where the initial wave propagation is directed towards the defect. We verified that it is appropriate that the integration in Eq. 3 is taken on the left of the defect (for positive velocities): other choices, such as integrating over the whole interval or only on the right of the defect, produce less clear and stable results, see panels (a), (b) and (c) in Fig. 3. Note that ${\mathfrak{D}}(t)$ is constructed to characterise the variance (and not the average) of the fluctuations of the density. Of course, if the defect is absent ${\mathfrak{D}}=0$.

**Figure 4:**
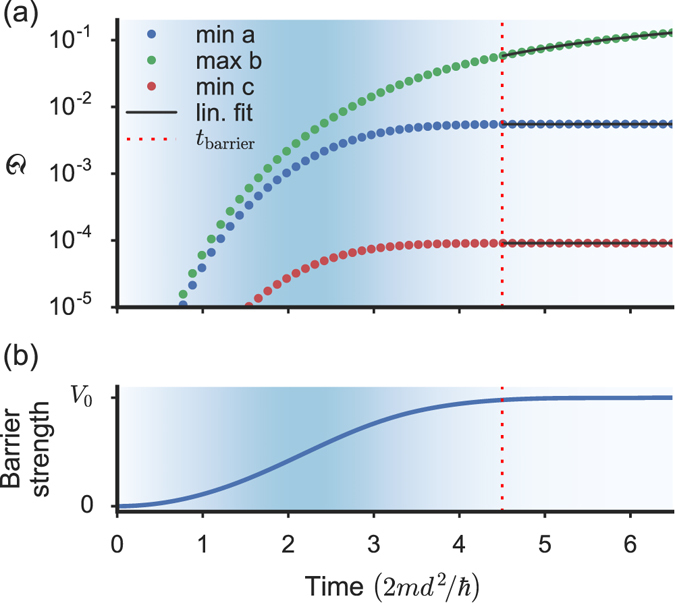
Suppressed excitation growth. (**a**) Disturbance ${\mathfrak{D}}$ calculated as a function of time for the initial velocities *v* corresponding to the minima and maximum labelled “a”, “b”, “c” in the main part of Fig. 3. (**b**) shows the corresponding time dependence of the barrier strength. The background shading indicates the rate of change of the barrier strength. For times *t* > *t*_barrier_, where the barrier strength has settled, ${\mathfrak{D}}$ has a linear dependence on time. The creation of excitations can then be characterised by the slope $\dot{{\mathfrak{D}}}$ of the disturbance at large times (the linear fits for the times considered in the calculation of $\dot{{\mathfrak{D}}}$ are plotted as a black lines) and it is extremely small at the velocities corresponding to the minima a and c (see central right panel in Fig. 6 for details).

For the numerical solution we use the projected fourth-order Runge-Kutta method in the interaction picture^37^, which we confirmed to be very stable for all times within the considered range. We made sure to choose a sufficiently large system size 2*L* and sufficiently short observation times to avoid finite size effects. For practical purposes we apply periodic boundary conditions on a domain with finite length $2L\gg d$ (with *x*∈[−*L*, *L*]) and only evaluate our disturbance estimator for times before any features emerging in the dynamics have reached the system boundaries. Up to that point no difference is found with respect to the homogeneous system for identical finite size systems with the same defect and a different choice of boundary conditions. We ensure that our results are never plotted for times beyond that limit. In fact our *L* is so large, up to *L* = 700 times the size of the defect, that we find a very long plateau, for example in the value of $\dot{{\mathfrak{D}}}(t)$ (see Fig. 4a), with stable and reliable numerical results before the limit imposed by the finite size of the simulation domain has any effect. In the following, we refer to this constant value reached by $\dot{{\mathfrak{D}}}(t)$ during the plateau simply as $\dot{{\mathfrak{D}}}$.

Given the explicit arguments of the Landau criterion it is important to clarify what is meant by completely restored superfluid flow above the critical velocity: (*i*) A supercritical solution of the dynamical equations exists for specific values of the superfluid’s momentum and system parameters (i.e. interaction strength and geometric properties of the defect) and the flow of such a solution is stable under small perturbations. (*ii*) The perturbations of the propagating flow created by the defect are bounded in time (at least for experimental time scales) and no new excitations are produced when the barrier is completely ramped on. (*iii*) The superfluid flow is physically inducible and accessible.

We have found that all three conditions are fulfilled for the supercritical flow discussed here. We have indeed verified that the production of excitations at the supercritical excitation-free points is bounded in time, that the flow is stable under small perturbations for times larger than the typical experimental time-scales of ultracold atom experiments with 1d Bose gases, and that the obtained findings do not crucially depend on the ramping time of the well/barrier.

## Results

It is well known that in the non-interacting regime the transmission coefficient across a square potential, as found by solving the time-independent *linear* Schrödinger equation, reaches exactly unity for specific values of the momentum of an incident plane wave (Ramsauer-Townsend resonances)^7^.

After the barrier has been ramped on, ${\mathfrak{D}}(t)$ is found to increase linearly with time both in the linear (*g* = 0) and in the nonlinear (*g* ≠ 0) case. In the linear case the behaviour of the rate of change of ${\mathfrak{D}}(t)$, i.e. $\dot{{\mathfrak{D}}}$, is qualitatively very similar to the behaviour of the reflection coefficient *R*, as shown in Fig. 2b, and both are vanishing near the points of perfect transmission: *R* is exactly zero there as obtained from a computation with the time-independent Schrödinger equation (and the barrier permanently on)^7^, while $\dot{{\mathfrak{D}}}$ in those points, as obtained from the time-dependent Schrödinger equation (and the barrier being ramped on), is found to be several order of magnitudes smaller than for other velocities. In other words, Fig. 2b also shows that the minima of $\dot{{\mathfrak{D}}}$ reproduce quantitatively the velocities for which the reflection is exactly zero in the time-independent computation.

Therefore we are led to take ${\mathfrak{D}}(t)$ also in the nonlinear case as a good indicator of the excitations produced by the barrier and $\dot{{\mathfrak{D}}}$ as a measure of the rate of the excitations produced. We observe that despite the general difficulty to define a transmission coefficient in the nonlinear case discussed above, a situation of flow unaffected by the defect can still be identified with perfect (unity) transmission, which is equivalent to no excitation production and therefore measured as $\dot{{\mathfrak{D}}}\approx 0$ in our case.

**Figure 5:**
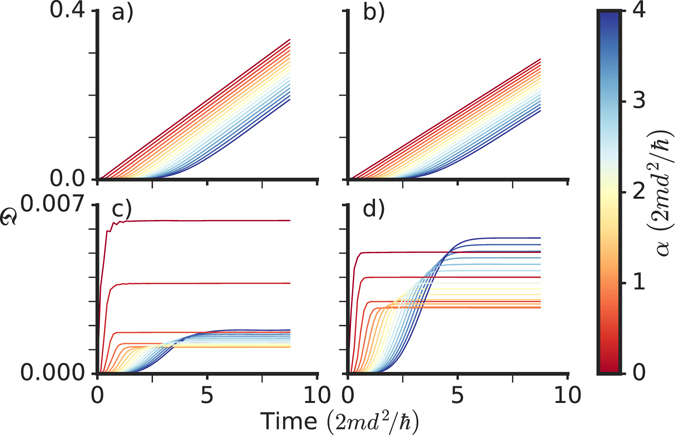
Effect of the ramping time on the excitation growth: disturbance as a function of time for varying ramping time constant *α*. After the barrier reaches its maximum value when *t* ~ *α*, the disturbance has a linear dependence on time. (**a**,**c**) Non-interacting (*gn*/*V*_0_ = 0) disturbance growth. (**b**,**d**) Disturbance growth for an interacting (*gn*/*V*_0_ = 7.5) gas. Top plots (**a**,**b**) show the behaviour for off-resonant momenta *kd* = 4.4 and *kd* = 5.4 while the bottom ones (**c**,**d**) are calculated at their respective values of resonant momenta *k*_res_. In the resonant case, both the absolute disturbance as well its asymptotic slope are orders of magnitude smaller than in the non-resonant case.

**Figure 6:**
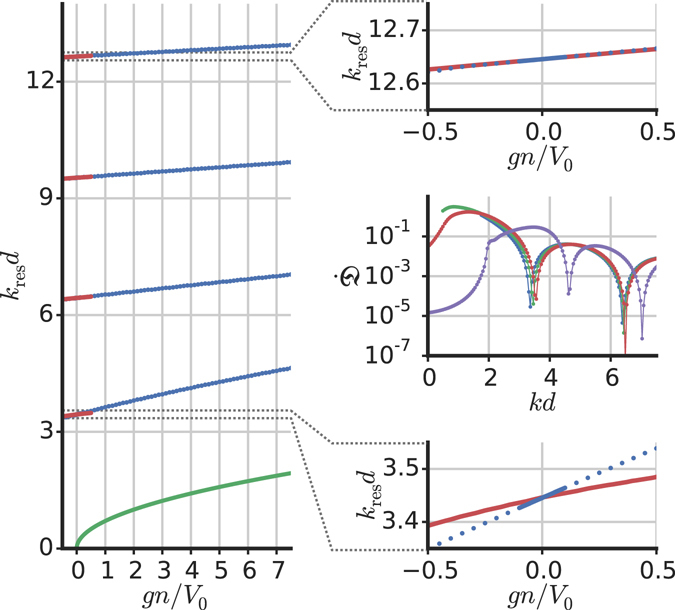
Resonant momenta. Centre-right: disturbance growth rate $\dot{{\mathfrak{D}}}$ as a function of *kd* for four different values of interaction: *gn*/*V*_0_ = −0.5, 0, 0.5, 7.5 (blue, green, red, purple lines, respectively). Left: Resonant momenta *k*_res_ *d* (with *mv*_res_ = *ħk*_res_) as a function of *gn*/*V*_0_ for the first four excitation-free points. The solid red lines are the perturbative prediction (Eq. 5), the blue points indicate numerically calculated values and the green line shows *k*_sound_ *d*, with *k*_sound_ = *mc*/*ħ* and the sound velocity $c=\sqrt{gn/m}$ (notice that in this figure we are fixing all parameters, including *V*_0_ = 2 × *ħ* ^2^/2*md* ^2^, and varying *g*). Bottom-right and top-right: detailed view of the first and fourth resonances, respectively.

Our results for the 1d case are summarised in Figs 3–6. Figure 3 shows that essentially no excitations are produced for velocities below the sound velocity $c=\sqrt{gn/m}$ (here *v*_*c*_ ≈ *v*_*L*_ = *c*). In 1d, the Landau criterion allows obtaining quantitatively the critical velocity and, as expected, excitations are produced for *v* > *v*_*c*_. Additionally, as time progresses ${\mathfrak{D}}(t)$ increases and, for large velocities, the growth rate of ${\mathfrak{D}}(t)$ is smaller. However, there are velocities *v* > *v*_*c*_, for which the production of excitations is inhibited. As shown in the inset of Fig. 3, close to these points the production of excitations is bounded in time. Panels (a), (b), (c) of the upper part of Fig. 3 show the density at a fixed time larger than *t*_barrier_. Away from the minima, phonons are emitted (Fig. 3b) while at the minima breathing states form inside the defect (Fig. 3a and Fig. 3c)^30^. We verified that these results depend neither on the particular measure of disturbance chosen, nor on the periodic boundary conditions, nor on the choice of *L*. These findings also do not critically depend on the value *α*. This is true for typical experimental values of *α* as well as for very small values of *α*, where small fluctuations of ${\mathfrak{D}}(t)$ occur for specific choices of quantifiers of the disturbance. We finally observe that our results hold for a wide range of interaction strengths, including large values of *g* > 0, so that the validity of these results spans from the regime where the healing length is smaller than the defect width to the regime where it is larger. For small negative values of *gn*/*V*_0_, in the interval between −0.5 and 0, the resonances are still present, as shown in Fig. 6. In this case, the time scale at which the attractive interaction causes instabilities that lead to a collapse is still larger than the simulation time, which is limited by the finite size of the domain. When *g* becomes more negative, then collapse and instabilities are observed.

As shown in Fig. 4a, there is a clear difference between the growth of ${\mathfrak{D}}(t)$ at the resonant and non-resonant velocities. At resonant velocities, excitations are produced exclusively during the ramping of the defect and the disturbance is afterwards constant. For non-resonant velocities the disturbance grows linearly with time, which leads us to quantify the growth of excitations by computing the time derivative of ${\mathfrak{D}}(t)$ for *t* > *t*_barrier_ (and checking that the obtained value does not depend on the computation interval). The analysis of the numerical results shows that $\dot{{\mathfrak{D}}}$ is extremely small at the resonant velocities (being suppressed by at least 4 orders of magnitude with respect to non-resonant velocities). $\dot{{\mathfrak{D}}}$ is also very small for *v* < *v*_*c*_ in agreement with the Landau criterion.

The creation of excitations can be well characterised by the slope of the disturbancefor *t* > *t*_barrier_, where ${\mathfrak{D}}(t)$ has a linear behaviour. In Fig. 5 we show ${\mathfrak{D}}(t)$ for *g* = 0 and for a finite value of *g*. In both cases the disturbance is evaluated for on- and off-resonance initial momentum. The asymptotic slopes $\dot{{\mathfrak{D}}}$ for off-resonance incident momenta are much larger than for the on-resonance momenta. For each of the cases we also vary the ramping time constant *α*, and it is seen that in all the cases–including the instantaneous ramp-on with *α* = 0–the behaviour of ${\mathfrak{D}}(t)$ is rather well approximated by a linear behaviour for times $\gtrsim \alpha$ and that the slope does not depend on the ramping time. We also observe that at the resonance points where the slope of ${\mathfrak{D}}(t)$ flattens, the behaviour of ${\mathfrak{D}}(t\gg {t}_{{\rm{barrier}}})$ is non-monotonous when *α* is increased. One can have a qualitative understanding of this by observing that–at the resonances–the disturbance ${\mathfrak{D}}(t)$ saturates asymptotically in time to a constant value when the wavelength inside the barrier resonates with the barrier width. This asymptotic value of ${\mathfrak{D}}(t)$ is minimal when the ratio between the crossing time $( \sim 2m{d}^{2}/\hslash )$ and the ramping time (~*α*) approaches a value on the order of unity. A barrier ramped too fast while being crossed by the particles produces a larger disturbance ${\mathfrak{D}}(t\gg {t}_{{\rm{barrier}}})$, as expected. Conversely, a barrier ramped up too slowly will produce a consistent non minimal disturbance ${\mathfrak{D}}(t\gg {t}_{{\rm{barrier}}})$ since the wavelength inside the barrier varies during ramping process and is not always resonant. Accordingly, the minimum for ${\mathfrak{D}}(t\gg {t}_{{\rm{barrier}}})$ must occur at an intermediate value of the (characteristic) ramping time *α*, comparable to the crossing time through the barrier, explaining the non-monotonous behavior displayed in Fig. 5 around *α* ~ 1 in the units of the figure.

Since we find that $\dot{{\mathfrak{D}}}$ is independent of the ramping time *α* both for vanishing and finite *g*, the resonances found in the linear case *g* = 0 must reproduce the momenta of the Ramsauer-Townsend effect, obtained by a time-independent computation in quantum mechanics textbooks^19, 20^. This result is confirmed in Fig. 6 where it is shown that for *g* = 0 the resonant momenta obtained by means of this dynamical method coincide with the results obtained by solving the time-independent linear Schrödinger equation. Since it is useful in the following, we note that the incident momenta *k*_res_ that result in Ramsauer-Townsend resonances, of unity transmission in the linear case (*g* = 0), are given by

4 $${k}_{{\rm{res}}}^{2}(g=\mathrm{0)}=\frac{2m{V}_{0}}{{\hslash }^{2}}+\frac{{n}^{2}{\pi }^{2}}{{d}^{2}},$$

with *n* = 1, 2, ….

In the central right inset of Fig. 6 we plot the values $\dot{{\mathfrak{D}}}$ as a function of the velocity for four interaction strengths. By performing the same analysis for different values of *gn*/*V*_0_, we obtain the behaviour of the resonant velocities *v*_res_ as a function of *g*. The results are plotted in the left part of Fig. 6 for the case of a barrier (positive *V*_0_) with *gn*/*V*_0_ varying between −0.5 and 7.5. In this figure the dimensionless parameters *k*_res_ *d* and *gn*/*V*_0_ are used, meaning that distances are measured in units of *d* and energies in units of *V*_0_. The presented results do not depend on the particular choice of *d* or *V*_0_ as long as those dimensionless parameters are used. Furthermore, by considering the slope $\dot{{\mathfrak{D}}}$ as a function of *k* for different values of *α*, we verified that within the achieved numerical precision, the value of the resonant momenta does not depend on *α*.

With respect to the non-interacting limit *g* = 0 the shift of the resonant momenta is positive for repulsive (*g* > 0) interactions, while it is negative for attractive (*g* < 0) interactions. Similar results hold for a potential well (*V*_0_ < 0), but with a negative shift for repulsive interactions and a positive one when the interactions are attractive. Our results are plotted in Fig. 6 together with a multiple-scale analytical derivation valid for small *g*, predicting values of *k*_barrier_ (defined below) corresponding to a total transmission across the barrier^38^. These resonant momenta are given by *k*_barrier_ *d* = *nπ* + *δ*, where ${k}_{{\rm{barrier}}}=\sqrt{{k}^{2}-\frac{2m{V}_{0}}{{\hslash }^{2}}}$ (with velocity *v* = *ħk*/*m* and *k* incident momentum). When there is no interaction (*g* = 0), then *δ* has to be zero and the linear resonant momenta (4) are recovered. In accordance with ref. 38 we find

5 $$\delta =\frac{3a(kd+n\pi )}{8},$$

with *a* = 2*mgn*/*v* ^2^. Note that our result (Eq. 5) differs from Eq. 24 of ref. 38 by the factor (−1)^*n*^ which is present there. The analytical predictions for small *g* match the numerical data well for the higher excitation-free points, but less so for the lower resonant velocities. The ratios of the slopes of *v*_res_ as a function of *g* between the analytical and the numerical results are 0.47, 0.83, 0.92 and 0.95 for the first four excitation-free points in increasing velocity order. To understand such a difference between our numerical findings and analytical results from multiple-scale analysis, we observe that in ref. 38 the computation is done with the time-independent nonlinear Schrödinger equation. This restricts the problem to static situations and requires a perturbative determination of the eigenfunctions with the defect permanently turned on. Here we instead study a dynamical problem in which the barrier is off at the beginning and then ramped on. This has two advantages: we are able to start from an exact eigenfunction (the plane wave), and we drive the system by adiabatically ramping the potential to the maximum of the resonance (while generically a multi-peak hysteretic structure of the values of transmission occurs when the nonlinear term is present). A possible reason of the mismatch between numerical and analytical results (also for very small *g*) could be attributed to the fact that our numerical procedure is based on the use of the full time-dependent Gross-Pitaevskii while the analytical computation is based on a perturbative treatment of the time-independent problem. However, as shown in Fig. 5, varying the ramping time *α*, and also bringing this time to zero, the slope $\dot{{\mathfrak{D}}}$ of the growth of the excitation does not vary. This happens also in the absence of interactions (*g* = 0) for which the slope has a clear minimum at the momenta given by the exact time-independent quantum mechanics computation^19, 20^. Given the fact that the same happens at finite *g* (see right part of Fig. 5), we conclude that the difference appears not to be due to ramping time effects. Since a perturbative computation should be exact by construction for small interactions, we believe that it would be interesting to obtain analytical and/or numerical solutions of the time-independent Gross-Pitaevskii equation in presence of a rectangular barrier and compare them both with the perturbative results^38^ and with the numerical results of the present paper.

### 2d square defect

**Figure 7:**
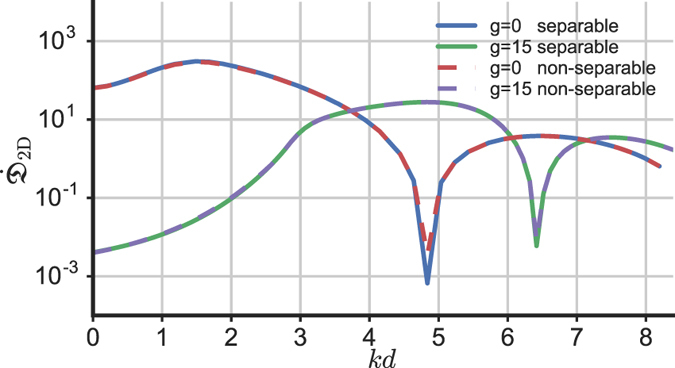
2d results. Rate of change of the disturbance defined in Eq. 7 as a function of the incident velocity *k* for different values of the interactions (*g* = 0 and *g* = 15) and of the parameter *ε*: separable stands for *ε* = 2 and the non-separable potential is with *ε* = 1. The initial wavefunction is $\psi (x,y,t=\mathrm{0)}={\psi }_{0}{e}^{ik(x+y)/\sqrt{2}}$.

To illustrate the robustness of the results presented in the previous section, we consider a 2d square defect described by the potential

6 $${V}_{2d}(x,y,t)=\{\begin{array}{cc}\varepsilon {V}_{0}\,\tanh ({t}^{{\scriptscriptstyle 2}}/{\alpha }^{{\scriptscriptstyle 2}}) & {\rm{f}}{\rm{o}}{\rm{r}}\,x\in [0,\,\,d]\,{\rm{a}}{\rm{n}}{\rm{d}}\,y\in [0,\,\,d]\\ {V}_{0}\,\tanh ({t}^{{\scriptscriptstyle 2}}/{\alpha }^{{\scriptscriptstyle 2}}) & {\rm{f}}{\rm{o}}{\rm{r}}\,x\in [0,d]\,{\rm{a}}{\rm{n}}{\rm{d}}\,y\notin [0,d]\\ {V}_{0}\,\tanh ({t}^{{\scriptscriptstyle 2}}/{\alpha }^{{\scriptscriptstyle 2}}) & {\rm{f}}{\rm{o}}{\rm{r}}\,x\notin [0,d]\,{\rm{a}}{\rm{n}}{\rm{d}}\,y\in [0,d]\\ 0 & {\rm{o}}{\rm{t}}{\rm{h}}{\rm{e}}{\rm{r}}{\rm{w}}{\rm{i}}{\rm{s}}{\rm{e}}\end{array},$$

where *ε* is a dimensionless parameter (see Fig. 2c,d). When *ε* = 2 the potential (Eq. 6) is separable and it can be written in the form *V*_2*d*_(*x*, *y*, *t*) ≡ *V*_1*d*_(*x*, *t*) + *V*_1*d*_(*y*, *t*) where *V*_1*d*_ is given by Eq. 2. Therefore, in the linear case, for *ε* = 2 fully transmitted incident velocities occur similarly to what happens in 1d, due to the separability of the problem. For *ε* ≠ 2 the potential 6 is not separable: nevertheless, both in the linear and in the nonlinear case we find a structure of peaks analogous to the 1d case with the disturbance pertinently defined as

7 $${{\mathfrak{D}}}_{2D}(t)={\int }_{-L}^{0}dx{\int }_{-L}^{0}dy{({|\psi (x,y,t)|}^{2}-{|\psi (x,y,t=0)|}^{2})}^{2}$$

(similar to the 1d case, other choices of ${{\mathfrak{D}}}_{2D}(t)$ display the peaks less clearly). Numerical results from the 2d Gross-Pitaevskii equation with the potential *V*_2*d*_ (Eq. 6) are reported in Fig. 7. We observe that the results do not sensibly depend on *ε* because the two potentials occupy an extended region and only differ in a small portion of it [0, *d*] × [0, *d*].

### Experimental Considerations

Superfluid motion in the presence of defects has been extensively investigated in ultracold atom experiments: superfluidity can be probed by stirring a laser beam^5, 39–41^ and the critical velocity has been measured^5^. Experiments on superfluid motion have been performed also with moving optical lattices^42^, in toroidal geometries^17, 18^, with ultracold fermions near unitarity^6^ and in two-dimensional Bose systems^43^.

An experimental setup to test the results presented in this paper can be implemented with ultracold Bose gases trapped on an atom chip. A 1d quasi condensate can fill a several hundred microns long potential tube^44^ with a very small potential variation along the tube and correspondingly homogeneous density. The gas can be set in motion at a controlled velocity by removing the residual longitudinal confinement and applying a short pulse of a magnetic gradient in the same direction. Velocities of the order of, or exceeding, a typical sound velocity of *c* ~ 1 mm/s can be achieved straightforwardly. By applying currents to microwires on the chip, a magnetic defect can be produced and controlled. Its geometric shape can be tailored with a resolution given by the atom-surface distance *z*_0_. In order for a rectangular defect with sharp edges to be formed, the defect length *d* must be a few times larger than *z*_0_. Here it is critical that *z*_0_ ≈ 1 so that individual excitationless resonances can be distinguished from the intermittent regimes of fast excitation growth. The excitation behaviour can be probed by varying the velocity *v* of the gas’s motion or by varying the final amplitude of the defect *V*_0_ at fixed *v*. The difference in *V*_0_ for the first two excitation-free points is expected to be ${\rm{\Delta }}{V}_{0}\approx \frac{3{h}^{2}}{2m}\frac{1}{{d}^{2}}=h\times 6.9\,{\rm{kHz}}\times {{\rm{\mu }}m}^{2}\frac{1}{{d}^{2}}$ for the example of ^87^Rb, so that $d\gtrsim {z}_{0}$ needs to be sufficiently small to maintain Δ*V*_0_ ≈ *μ* ≈ *h* × 1 kHz, where *μ* is the chemical potential of the repulsively interacting gas. Appropriate ramping times of the defect are of the order of milliseconds (*t*_barrier_ ≈ 40 ms) for *d* ≈ 3 μm.

## Conclusions

We have studied the propagation of matter waves across defects of rectangular shape in 1d and 2d starting from a stationary flow solution with velocity *v* in the defect-free case and then ramping on the defect. For velocities smaller than a critical velocity *v*_*c*_ there is no production of excitations (with *v*_*c*_ very well approximated by the Landau critical velocity *v*_*L*_ in the 1d case). For a set of arbitrarily large supercritical velocities *v* > *v*_*c*_ the growth of excitations is fully suppressed, contrary to the generic expectation based on the Landau criterion. For these velocities, we find the production of excitations to be bounded in time and to stop entirely when the defect is completely turned on. Such excitation-free supercritical velocities are present both for wells and barriers, and for repulsive and (small) attractive interactions. We observe that even though in the nonlinear case bound states and bifurcation effects are expected, our protocol of ramping on the defect allows us to access the excitation-free points in a clean way, not depending on the ramping time.

The obtained excitation-free supercritical velocities are the nonlinear counterpart of the velocities having total transmission in the linear Schrödinger case (Ramsauer-Townsend resonances) and are due to the resonance between the length scale associated with the matter wave momentum (~2*π*/*k*) and the length scale of the defect. The shift from the resonance is positive (negative) for repulsive (attractive) interaction in the case of barrier defects, and vice versa for well defects. We expect that such excitation-free supercritical velocities exist for a wide range of barrier shapes characterised by a well defined length scale, e.g. for trapezoidal defects or two delta-peaked potentials. The steeper the defect is at its edges, the more robust will the inhibition of excitations be in the vicinity of a set of supercritical velocities. We verified that our results are not a specific features of one-dimensionality by showing that similar findings are obtained in a 2d geometry. We also expect that the phenomenon may occur in a fermionic superfluid, but of course further studied in this directions are needed.

Previous studies^10, 13, 14^ addressed superfluid flow for *v* > *v*_*c*_, implying the existence of a second larger critical velocity ${v}_{c}^{\ast } > {v}_{c}$ with the possibility of *partial* superfluidity (0 < *ρ*_*s*_ < *ρ* for ${v}_{c} < v < {v}_{c}^{\ast }$). Here, in contrast, we show that *total* transmission, and therefore *perfect* superfluidity (*ρ*_*s*_ = *ρ*), can occur for *v* > *v*_*c*_ for specific shapes of defects. Moreover (perfect) superfluidity persists for a series of specific, *arbitrarily large* values of the incident velocity. These findings are independent of whether or not the Landau critical velocity coincides with *v*_*c*_ or ${v}_{c}^{\ast }$.

We expect that our results are also obtained for other shapes of the defects also characterized by a single length scale, as is the case with two *δ* potentials separated by a distance *d*. When this length resonates with the incident wavelength, perfect transmission and complete superfluidity are restored, the key mechanism being the resonance between the non-linear wave propagation of the superfluid and the defect. A general potential not characterized by a single length scale will typically not exhibit  the resonances discussed in the present paper and, in particular, even when only two length scales are involved, transmission resonances will only be present if the lengths are commensurate.

We note that in our treatment we did not explicitly include dissipation. In general the time scale at which superfluidity is destroyed due to dissipation may depend on the incident velocity. However, for typical experimental parameters, dissipation only plays a significant role at time scales longer than our simulation times and therefore our results should hold even in the presence of dissipation. Nonetheless, in future work it would be interesting to include sources of dissipation in the presence of defects characterised by a single length scale.

Understanding matter-wave propagation in the presence of tailored defect potentials is important in a variety of applications, ranging from quantum technology applications, in particular quantum sensors based on matterwave interferometry^45^, to fundamental studies such as the study of analogue gravity models and acoustic Hawking radiation in Bose-Einstein condensates^46^.

Motivated by our work, it will be interesting to study general criteria for the existence and stability of supercritical solutions of the nonlinear Schrödinger equation in the presence of defects with a well-defined length scale. Finding exact solutions would ultimately remove the residual numerical uncertainty in excitation growth at the supercritical velocities with total transmission. Moreover, intriguing possibilities arise for utilising supercritical points in measurement devices based on superfluids and superconductors. Tuning a barrier to a supercritical flow resonance would facilitate precise determination of unknown external parameters affecting the flow velocity, such as rotation, and performing selective measurements at supercritical velocities.
